# Increased Tartrate-Resistant Acid Phosphatase Expression in Osteoblasts and Osteocytes in Experimental Osteoporosis in Rats

**DOI:** 10.1007/s00223-013-9834-3

**Published:** 2014-01-07

**Authors:** Lene B. Solberg, Sverre-Henning Brorson, Gunhild A. Stordalen, Espen S. Bækkevold, Göran Andersson, Finn P. Reinholt

**Affiliations:** 1 Department of Pathology, Oslo University Hospital, Rikshospitalet, Postbox 4950, Nydalen, 0424 Oslo, Norway; 2 Division of Pathology, F46, Department of Laboratory Medicine, Karolinska Institutet, Karolinska University Hospital Huddinge, Huddinge, 141 86 Stockholm, Sweden

**Keywords:** TRAP, Osteoblast, Osteocyte, Ovariectomy, Vitamin D

## Abstract

Tartrate-resistant acid phosphatase (TRAP) is known as an osteoclast marker, but osteoblasts and osteocytes in the vicinity of bone remodeling sites also express TRAP. Cell culture studies suggest that osteoblasts endocytose osteoclastic TRAP for inactivation. To evaluate whether changes in osteoclast activity could alter TRAP expression in osteoblasts and/or osteocytes in vivo, we studied the ovariectomized and vitamin D-deficient rat (Ovx-D) and rats healing from rickets. Bone sections were analyzed for TRAP gene expression by in situ hybridization, TRAP protein by immunogold labeling, and TRAP enzyme activity using the fluorescent substrate ELF97. Osteoblasts and osteocytes close to intracortical remodeling sites and bone surfaces demonstrated TRAP, most prominently in cancellous bone and osteocytes. Intracellular TRAP was located to electron-dense vesicles with similar morphology in both cell types. Ovx-D increased osteoclast activity (*p* < 0.001) and ELF97^+^ osteocytes (*p* < 0.05) in cancellous bone, but no corresponding increase was observed in the osteocyte lacunar area. The level of TRAP^+^ vesicles in cortical osteoblasts (*p* < 0.01) in Ovx-D rats was also increased. Enhanced osteoclast activity was noted in healing rickets after 72 h (*p* < 0.05), but no differences in TRAP expression were detected in osteoblasts or osteocytes. Thus, increased osteoclast activity does not affect TRAP expression in osteoblasts and osteocytes, favoring the notion that increased TRAP in these cells is rather due to increased synthesis. Although the role of TRAP in osteoblasts and osteocytes remains elusive, we speculate that the function is related to the capability of the enzyme to regulate the phosphorylation of proteins known to be expressed by these cells.

**Electronic supplementary material:**

The online version of this article (doi:10.1007/s00223-013-9834-3) contains supplementary material, which is available to authorized users.

## Introduction

Tartrate-resistant acid phosphatase (TRAP; ACP5, EC 3.1.3.2)—also known as purple acid phosphatase, uteroferrin, or type 5 acid phosphatase [1]—has been an established marker for osteoclasts and bone resorption for more than 50 years. TRAP is synthesized as a relatively inactive proenzyme (monomeric TRAP [mTRAP], loop-TRAP, serum TRAP 5a), and proteolytic cleavage by members of the cathepsin family or other proteinases increases the catalytic activity at least tenfold [2, 3]. Cleaved, active TRAP is identical to osteoclastic TRAP and serum TRAP 5b [4] and is able to dephosphorylate bone matrix proteins, e.g., osteopontin (OPN) and integrin binding sialoprotein (IBSP) [5, 6], as well as to generate reactive oxygen species for bone matrix degradation [7]. Halleen et al. [8] have shown that the serum activity of TRAP 5b is significantly elevated in patients with osteoporosis and negatively correlated with bone mineral density (BMD). Studies on mice have yielded similar results: global deletion of TRAP leads to disturbed endochondral ossification and a mild osteopetrotic phenotype [9, 10], while overexpression of TRAP results in enhanced bone turnover and a mild osteoporotic phenotype [11]. In addition to osteoclasts, TRAP has been reported in osteoblasts and osteocytes [12–21] closely related to bone surfaces [12, 21] or intracortical remodeling sites [12, 16] in rat bone tissue. The origin and function of TRAP in these cells have been debated; one hypothesis is that osteoclastic TRAP from the resorption lacunae is endocytosed by the osteoblasts and/or osteocytes. This theory is supported by cell culture studies reporting that osteoblast-like cells are able to engulf osteoclastic TRAP and inactivate the enzyme, suggesting that this could control the enzyme activity and prevent further degradation of matrix constituents [17, 18]. However, endogenous TRAP synthesis has been demonstrated in osteoblasts [21] and in osteocytes [16] in areas close to bone-resorbing osteoclasts, suggesting that TRAP may take part in mechanisms controlling the direction of osteoclastic bone resorption [16]. Qing and coworkers [19] have demonstrated enlarged osteocyte lacunae and canaliculi and increased amounts of TRAP and cathepsin K in osteocytes in lactating mice, suggesting that osteocytes are able to remodel their own matrix environment through osteoclast-like mechanisms under specific conditions.

To increase the knowledge of TRAP in osteoblasts and osteocytes, we analyzed two experimental rat models with disturbed bone metabolism to investigate whether changes in osteoclast activity could alter TRAP protein expression and enzyme activity in osteoblasts and/or osteocytes in vivo. The ovariectomized and vitamin D-depleted rat (Ovx-D) mimics human osteoporosis as seen in elderly postmenopausal women with reduced BMD of metaphyseal bone [22]. Osteoclast activity and TRAP protein expression in osteoblasts and TRAP activity in osteocytes were increased in Ovx-D rats. Moreover, Ovx-D rats presented a tendency toward increased TRAP mRNA expression in osteocytes, questioning the hypothesis of endocytosis being the mechanism enhancing TRAP protein expression and enzyme activity in osteoblasts and osteocytes in these rats. To further address this question, rats healing from nutritionally induced low-phosphate and vitamin D-deficiency rickets (experimental rickets) were analyzed as a model of increased osteoclast activity [23]. However, such rats did not show any differences in the level of TRAP protein expression or enzyme activity either in osteoblasts or in osteocytes, making it less likely that osteoblasts and osteocytes endocytose osteoclastic TRAP.

## Materials and Methods

All analyses were performed on coded sections using AnalySIS FIVE (Olympus Soft Imaging Solutions, Münster, Germany) following the suggestions for standardized nomenclature from the American Society for Bone and Mineral Research [24].

### Animal Experiments

The Guide for the Care and Use of Laboratory Animals [25] was followed, and the study protocols were approved by the Norwegian National Animal Research Authority. The Ovx-D model has been reported in detail previously [22]. Low-phosphate and vitamin D-deficiency rickets and healing for 48 and 72 h were induced as described by Hollberg et al. [23]. Blood was sampled from the animals, and their tissues were fixed by vascular perfusion [22] at the end of the experiments.

### Tissue Preparation

Femurs and tibias were dissected free, immersed in 2 % phosphate-buffered paraformaldehyde, and decalcified in 7 % EDTA with 0.5 % paraformaldehyde for 40 days. Bone tissues for light microscopy or fluorescence microscopy were paraffin-embedded, and 2–3-μm-thick sections were cut, picked up on glass slides, and rehydrated through a series of graded alcohols. Bone tissues for transmission electron microscopy (TEM) were cut into small samples (~1 mm^3^), fixed in 2 % paraformaldehyde and 0.5 % glutaraldehyde, and embedded with progressive lowering of temperature (Leica EM AFS; Leica Microsystems, Wetzlar, Germany) in the acrylate- and methacrylate-based resin Lowicryl HM23 according to our established protocol [26]. Ultrathin sections (75 nm) were mounted on formvar-coated nickel slot grids.

### Osteoclast Activity

Total numbers of osteoclasts relative to tissue volume (N.Oc/TV) and osteoclast surface relative to bone surface (Oc.S/BS) were estimated by point counting in a squared grid within 500 μm from the epiphyseal/metaphyseal border (EMB) at TEM micrographs. An osteoclast was defined as a multinuclear cell attached to a bone surface or in the intertrabecular space with characteristics such as ruffled border, intracytoplasmic vesicles, and abundant mitochondrial profiles. Twenty micrographs from each animal were analyzed, and the ratio (Oc.S/BS)/(N.Oc/TV) was calculated for each animal and compared between Ovx-D and sham as a parameter of osteoclast activity [27]. In the experimental rickets group, commercially available kits were used for determination of osteoclast-derived C-telopeptide fragments of collagen type I (CTX) (RatLaps™ EIA; Immunodiagnostic Systems, Tyne and Wear, UK) and osteoclast-derived TRAP 5b (RatTRAP™ Assay, Immunodiagnostic Systems). Serum was analyzed in all animals, and the CTX/TRAP 5b ratio, as a parameter for osteoclast activity [27], was calculated for each animal and compared between the groups.

### In Situ Hybridization

TRAP gene expression was studied by in situ hybridization. A gene sequence for rat TRAP [28] was amplified by conventional PCR using cDNA from rat bone and oligonucleotide forward and reverse primers: rnTRAP.for 5′-ACGCCAATGACAAGAGGTTC-3′, rnTRAP.rev 5′-ACATAGCCCACACCGTTCTC-3′ (Life Technologies, Carlsbad, CA, USA) and cloned in a Dual Promoter TA Cloning Kit (Life Technologies). The cloned insert was sequenced to establish the orientation (Seqlab, Göttingen, Germany). A digoxigenin (DIG)–conjugated complementary RNA probe was synthesized using T7 or Sp6 polymerase to yield the probe in the sense or antisense direction (DIG-labeling kit; Roche Diagnostics, Oslo, Norway). Longitudinal sections from the tibial diaphysis (Ovx-D/sham) and femoral diaphysis (experimental rickets) were subjected to hybridization following our established protocol [29]. TRAP mRNA^+^ osteocytes were quantified in cortical bone within 4–10 mm from the proximal EMB by point counting in a squared grid. Three sections were examined from each animal and their means compared between the groups. Tibial diaphyses were examined twice with an interclass correlation of *p* < 0.001 and Cronbach’s alfa of 0.94. Staining of osteoclasts from the femoral metaphysis in healing for 72 h was used as a positive control. The sense probe did not show any staining.

### Immunofluorescence

To estimate TRAP enzyme activity and the putative colocalization of the translated mTRAP protein and the enzyme activity, fluorescence-based staining with rabbit anti-mTRAP and ELF97 was performed. With low pH (<6.0) ELF97 is cleaved by activated acid phosphatase, yielding a bright yellow–green fluorescence precipitate [30, 31]. Rabbit anti-mTRAP was the same as previously used [32]. The ELF97 Endogenous Phosphatase Detection kit, AlexaFlour555 conjugated secondary antibody, and DAPI Nucleic Acid Stain were purchased from Molecular Probes (Eugene, OR, USA). Longitudinal sections from distal femoral metaphysis and diaphysis were analyzed. Images were obtained by Nikon DS-Fi2 color camera (Nikon Instruments, Melville, NY, USA) using UV and Cy3 filters and added in ImageJ [33]. ELF97^+^ osteocytes (Ot), mTRAP^+^ Ot, ELF97mTRAP^+^ Ot, and total Ot were quantified in cancellous bone within 1 mm into the metaphysis from the proximal EMB and in cortical bone within 4–10 mm from the proximal EMB. The means were calculated for each animal with respect to the parameters above and used for comparison between the groups. Nonspecific rabbit IgG served as a negative control for mTRAP, while TRAP enzyme was inactivated using 100 μM molybdate before adding ELF97 to evaluate the background fluorescence.

### Immunogold Labeling for TEM

To evaluate the distribution of TRAP in osteoblasts and osteocytes, bone sections from the tibial diaphysis (Ovx-D/sham) and the proximal tibial metaphysis and diaphysis (experimental rickets) were labeled with rabbit anti-TRAP (SB-TR103, Immunodiagnostic Systems). Immunogold labeling was performed as earlier described [34]. Nonspecific rabbit IgG served as a negative control. Micrographs from 10–20 osteoblasts and osteocytes were randomly sampled from each animal. An osteoblast was defined as a mononuclear cell attached to osteoid with prominent endoplasmatic reticulum (ER) and Golgi complexes. An osteocyte was defined as a mononuclear cell embedded in bone matrix with characteristic canaliculi. A TRAP^+^ vesicle was defined as a vesicle of moderate electron density containing four or more gold particles. An area of TRAP^+^ vesicles (TRAPv.Ar) relative to the area of cytoplasm (Cy.Ar) was measured in each osteoblast and osteocyte and the mean of the ratios (TRAPv.Ar/Cy.Ar) for each animal compared between the groups. Cells were analyzed twice with respect to TRAPv.Ar/Cy.Ar with an interclass correlation of *p* < 0.001 and Cronbach’s alfa of 0.98. Large standard deviations (SDs) in the ratios were observed for both osteoblasts and osteocytes within the Ovx-D and sham groups. To elucidate whether this phenomenon was due to differences between the animals in each group or in each animal, eight bone levels in one animal from each group were examined. The results demonstrated that the large SDs between the animals corresponded to the SDs between bone levels in each animal, indicating large biological variation (data not shown).

### Measurements of the Extent of TRAP^+^ Osteocytes

Semiquantitative measurements were performed on sections subjected to in situ hybridization or immunofluorescence in order to estimate the distance from bone surfaces or bone remodeling surfaces toward osteocytes expressing TRAP mRNA, TRAP protein (mTRAP), or TRAP enzyme activity in cancellous and cortical bone. All animals subjected to TRAP mRNA in situ hybridization as well as three animals from each group in both cancellous and cortical bone subjected to fluorescence-based staining with mTRAP and the fluorescence substrate ELF97 were analyzed.

### Osteocyte Lacunar Area

Longitudinal tibia mid-diaphyseal sections from Ovx-D and sham animals at the same bone level were subjected to conventional hematoxylin–eosin–saffron (HES) staining. The osteocyte lacunar area was measured within 1 mm at three discrete sites separated by 1 mm in a cross-sectional manner. Both cortices were included, and 200–250 osteocytes were measured per animal. The means of the osteocyte lacunar area were calculated and compared between the groups.

### Statistics

Statistical analyses were performed in PASW Statistics 18 (SPSS, Inc., Chicago, IL, USA). Parametric tests were used to compare the measured data, Student’s *t* test for two variables and one-way analyses of variance (ANOVA) for *k* variables. Nonparametric tests, Mann–Whitney for two variables, and Kruskal–Wallis for *k* variables were applied to the semiquantitative data. A *p* value of 0.05 was considered significant in all tests.

## Results

### Animal Models

Ovx-D rats developed osteoporosis with reduced trabecular bone volume (BV/TV) in the femoral head, *p* < 0.001, and decreased BMD in the femoral neck and the lower lumbar vertebra, *p* < 0.001 (Online Resource 1a, b) as well as undetectable serum levels of 25(OH)D and serum estradiol within the human postmenopausal range [22]. Low-phosphate and vitamin D-deficiency rickets with characteristic morphological changes (Online Resource 1c) were in line with previous experience with the model [23].

### Enhanced Osteoclast Activity in Animal Models

**Fig. 1:**
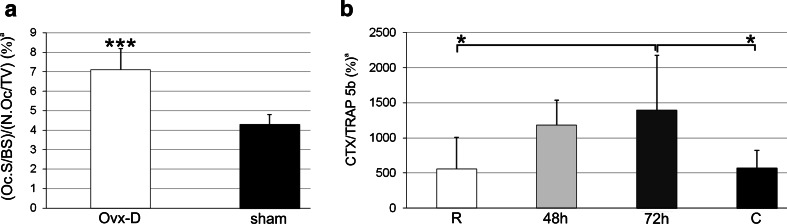
Osteoclast activity. **a** The ratio of (Oc.S/BS)/(N.Oc/TV) in cancellous bone was increased in Ovx-D versus sham (Student’s *t* test, *n* = 7 for both). **b** The ratio of serum CTX/TRAP 5b was increased in healing for 72 h compared with fulminant rickets (*R*) and controls (*C*) (ANOVA, *n* = 7 for all). ^a^Results are presented with mean and SD, *p < 0.05, ***p < 0.001

To evaluate TRAP positivity in osteocytes and osteoblasts in relation to osteoclast activity, we calculated osteoclast activity as Oc.S/BS relative to N.Oc/TV and CTX relative to TRAP 5b in serum in Ovx-D/sham and experimental rickets, respectively. Increased osteoclast activity was observed in Ovx-D versus sham (Fig. 1a) as well as in healing rickets after 72 h compared to fulminant rickets and normal controls, reflecting the healing of the growth plate with enhanced resorption monitored by an increased CTX/TRAP 5b ratio in serum (Fig. 1b).

### TRAP is Increased in Osteoblasts and Osteocytes in Ovx-D

**Fig. 2:**
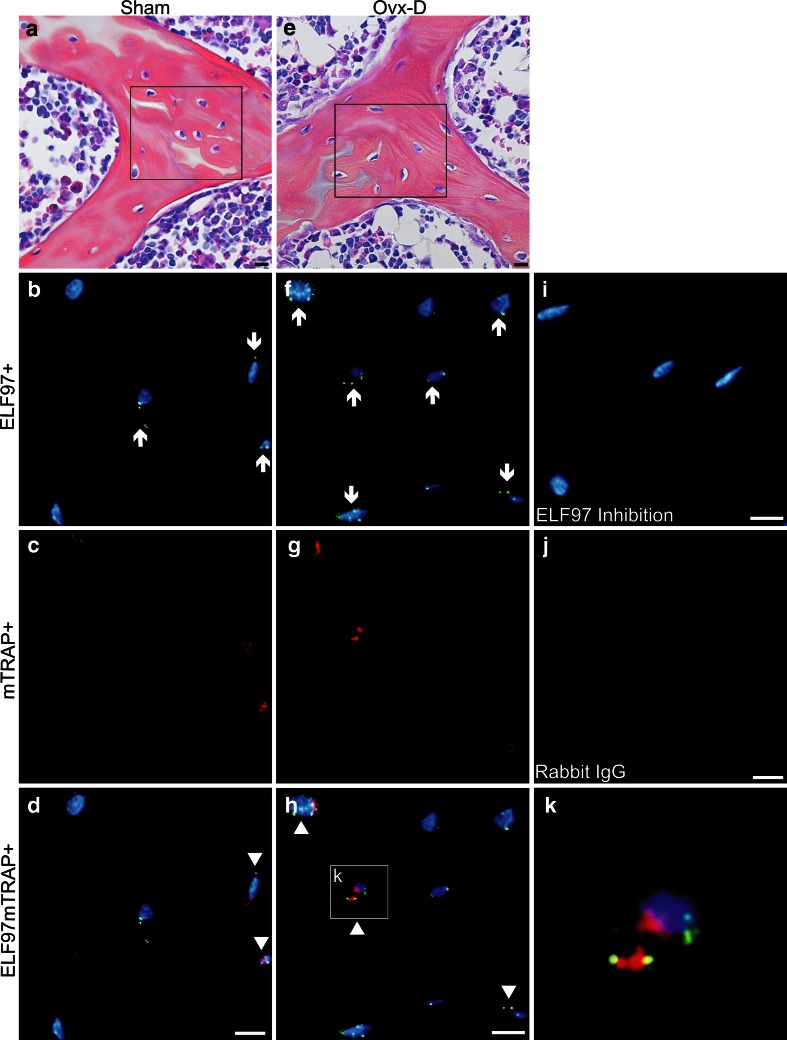
TRAP enzyme activity and monomeric TRAP (mTRAP) immunolabeling in osteocytes (Ot) in cancellous bone from distal femoral metaphysis. HES stained sections show the tissue architecture of cancellous bone in **a** sham and **e** Ovx-D. *Boxes* indicate areas corresponding to the immunofluorescence images. ELF97^+^ Ot (*yellow*-*green*, *arrows*), mTRAP^+^ Ot (*red*), and ELF97mTRAP^+^ Ot (*arrowheads*) in **b–d** sham and **f–h** Ovx-D. **i** Inhibition of TRAP enzyme activity with molybdate demonstrated low background fluorescence for ELF97. **j** Unspecific rabbit IgG served as negative control for mTRAP with low background fluorescence. **k** A high-power image shows the staining of ELF97 and mTRAP in osteocytes. *Scale bars* 10 μm

**Fig. 3:**
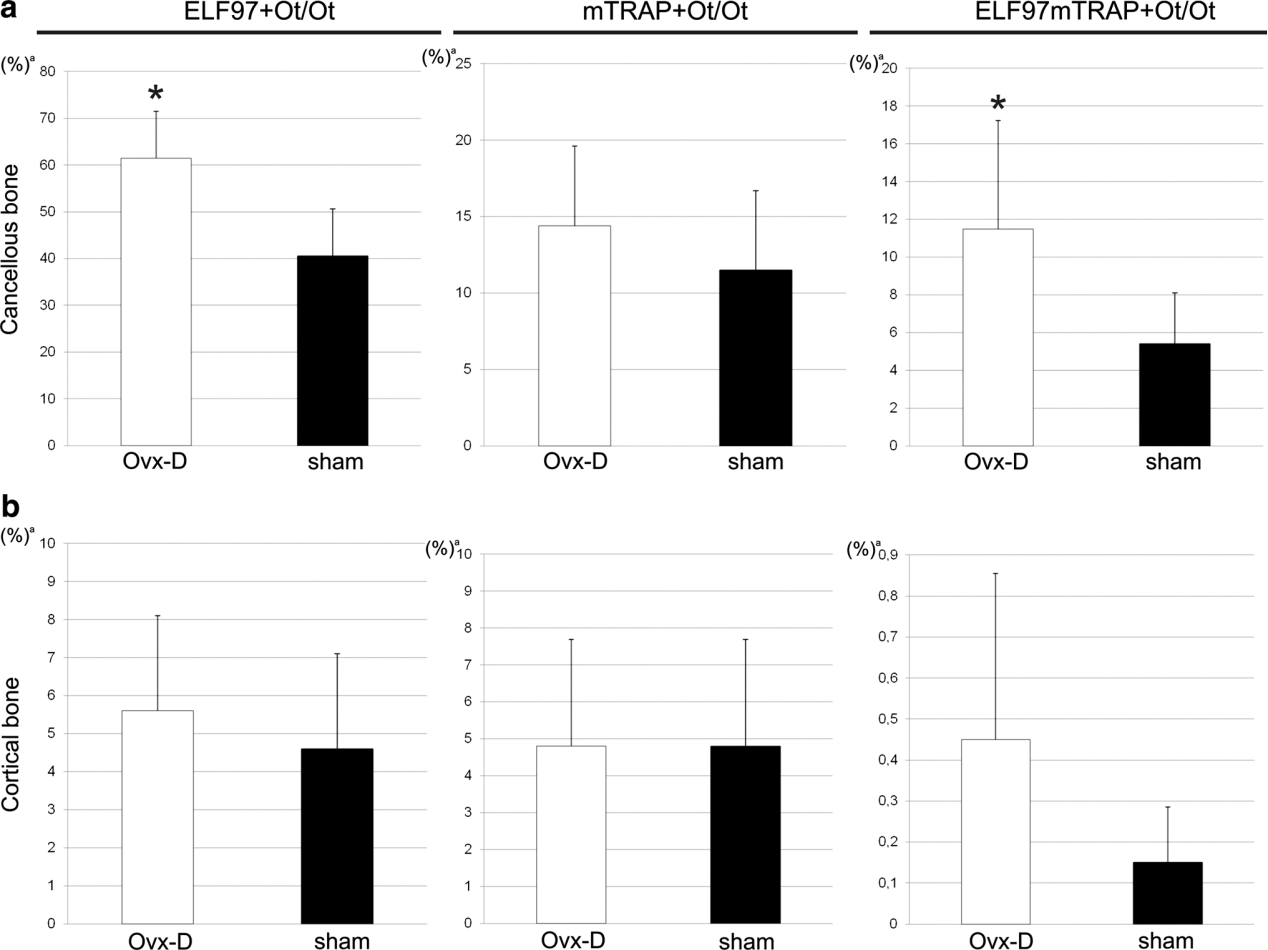
TRAP enzyme activity in osteocytes in cancellous and cortical bone in the experimental osteoporosis model. **a** ELF97^+^ Ot/Ot and ELF97mTRAP^+^ Ot/Ot were significantly increased in Ovx-D versus sham in cancellous bone, **b** while there were no significant differences between the groups in cortical bone (Mann–Whitney test, *n* = 7 for both). ^a^Results are presented with mean and SD, *p < 0.05

**Fig. 4:**
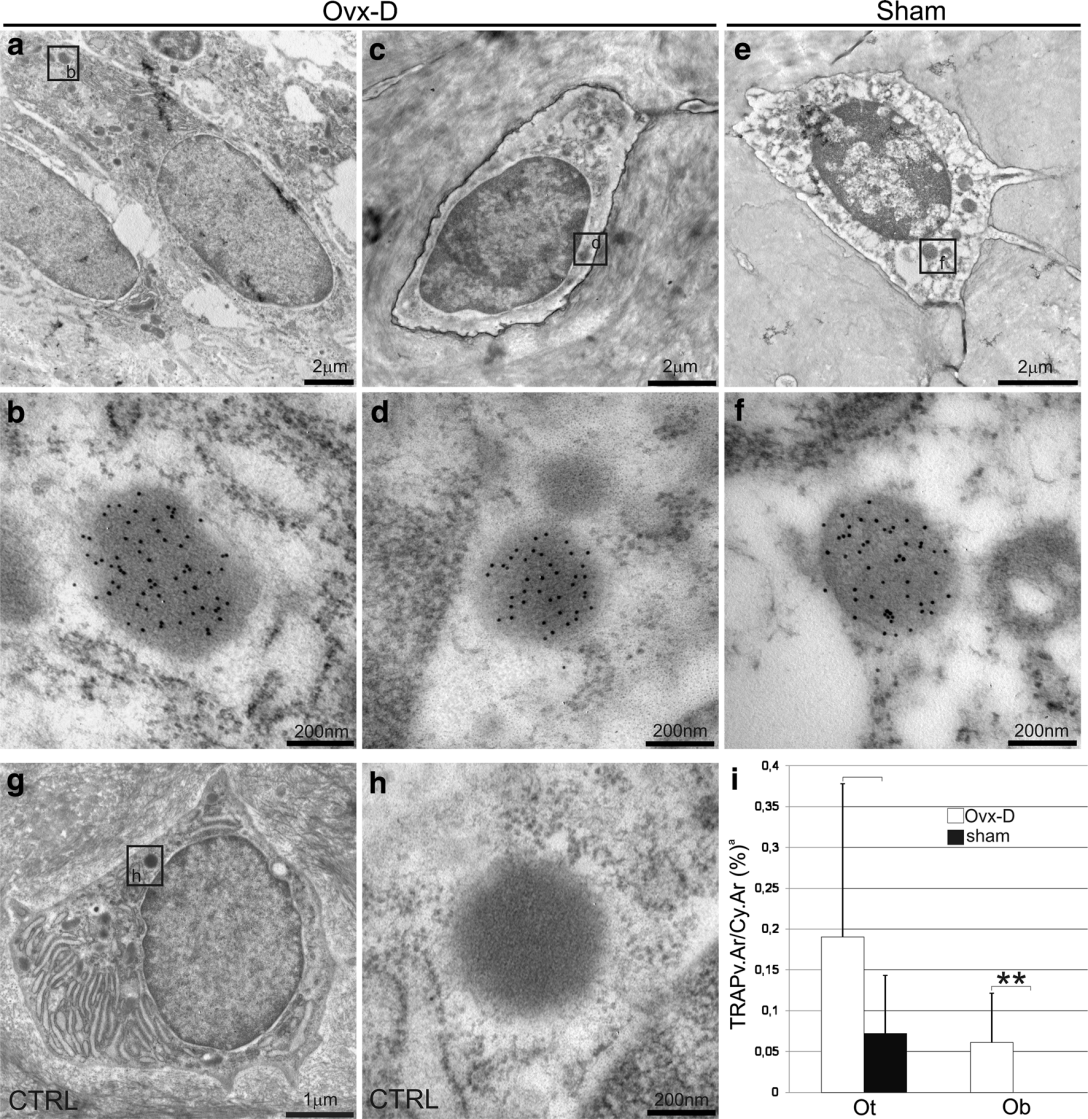
TEM micrographs from tibial diaphysis in Ovx-D and sham. Images with overview and close-ups show examples of immunogold labeling for TRAP in intracellular vesicles in osteoblasts (*Ob*) and osteocytes (*Ot*) in cortical bone: **a**, **b** osteocyte and **c**, **d** osteoblast from Ovx-D and **e**, **f** osteocyte from sham with TRAP^+^ vesicles. **g**, **h** Unspecific rabbit IgG served as negative control and did not label the vesicles in Ovx-D. **i** Significantly increased TRAP vesicle area versus area of cytoplasm (TRAPv.Ar/Cy.Ar) ratio in osteoblasts in Ovx-D versus sham (Mann–Whitney test, *n* = 7 for both). ^a^ Results are presented with mean and SD, **p < 0.01

**Fig. 5:**
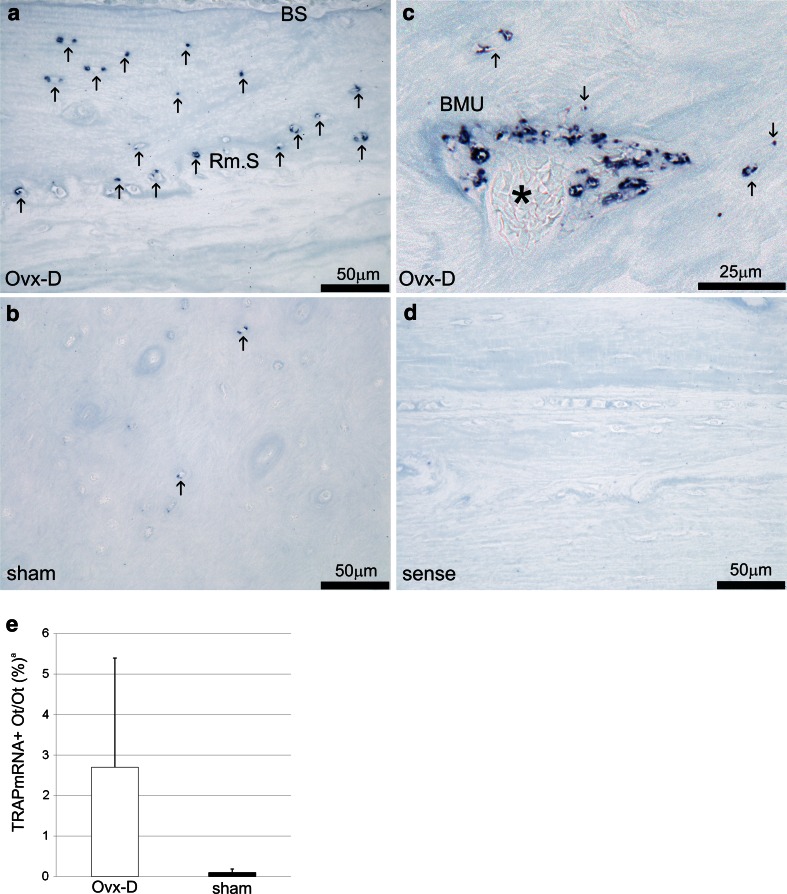
In situ hybridization for TRAP mRNA in tibial diaphysis in Ovx-D and sham animals. **a** TRAP mRNA^+^ osteocytes (*Ot*, *arrows*) were stained dark blue and observed to be closely related to the bone surface (*BS*) and the bone remodeling surface (*Rm.S*) in Ovx-D. **b** Sham animals demonstrated a limited number of TRAP mRNA^+^ osteocytes (*arrows*) **c** The method was confirmed by positive staining of an osteoclast in a bone morphogenic unit (*BMU*) with TRAP mRNA^+^ osteocytes (*arrows*) in close vicinity (*Ovx-D*). *Central capillary. **d** The sense probe displayed no staining and served as a negative control. **e** There was no significant difference in TRAPmRNA^+^ Ot/ Ot between Ovx-D and sham (Mann–Whitney test, *n* = 7 for both). ^a^ Results are presented with mean and SD

In cancellous bone ELF97^+^ Ot/Ot and colocalized ELF97mTRAP^+^ Ot/Ot were increased in Ovx-D versus sham (Figs. 2, 3a). Ovx-D also demonstrated a tendency to increase in ELF97^+^ Ot/Ot and ELF97mTRAP^+^ Ot/Ot versus sham in cortical bone (Online Resource 2, Fig. 3b). TEM analyses showed TRAP in intracellular electron-dense vesicles (200–500 nm in diameter) with similar morphological features in both osteoblasts and osteocytes in cortical bone. However, no general pattern of location in the cytoplasm was detected, and we were not able to demonstrate any fusion between TRAP^+^ vesicles and the cell membrane or any coated pits at the cell surface containing TRAP (Fig. 4a–h). Semiquantitative measurements of the area of TRAP^+^ vesicles relative to total cytoplasmic area (TRAPv.Ar/Cy.Ar) showed an increased ratio in osteoblasts and osteocytes in Ovx-D compared with sham, significant in osteoblasts (Fig. 4i). In situ hybridization demonstrated TRAP mRNA in osteocytes in cortical bone close to bone surface and intracortical remodeling sites in both Ovx-D and sham animals (Fig. 5a–c). However, only a small proportion of osteocytes in cortical bone were TRAP mRNA^+^: 2.9 % in Ovx-D versus 0.09 % in sham. Although the difference appeared striking, the result was not statistically significant (Fig. 5e).

### Healing from Rickets Does Not Alter TRAP in Osteoblasts or Osteocytes

**Fig. 6:**
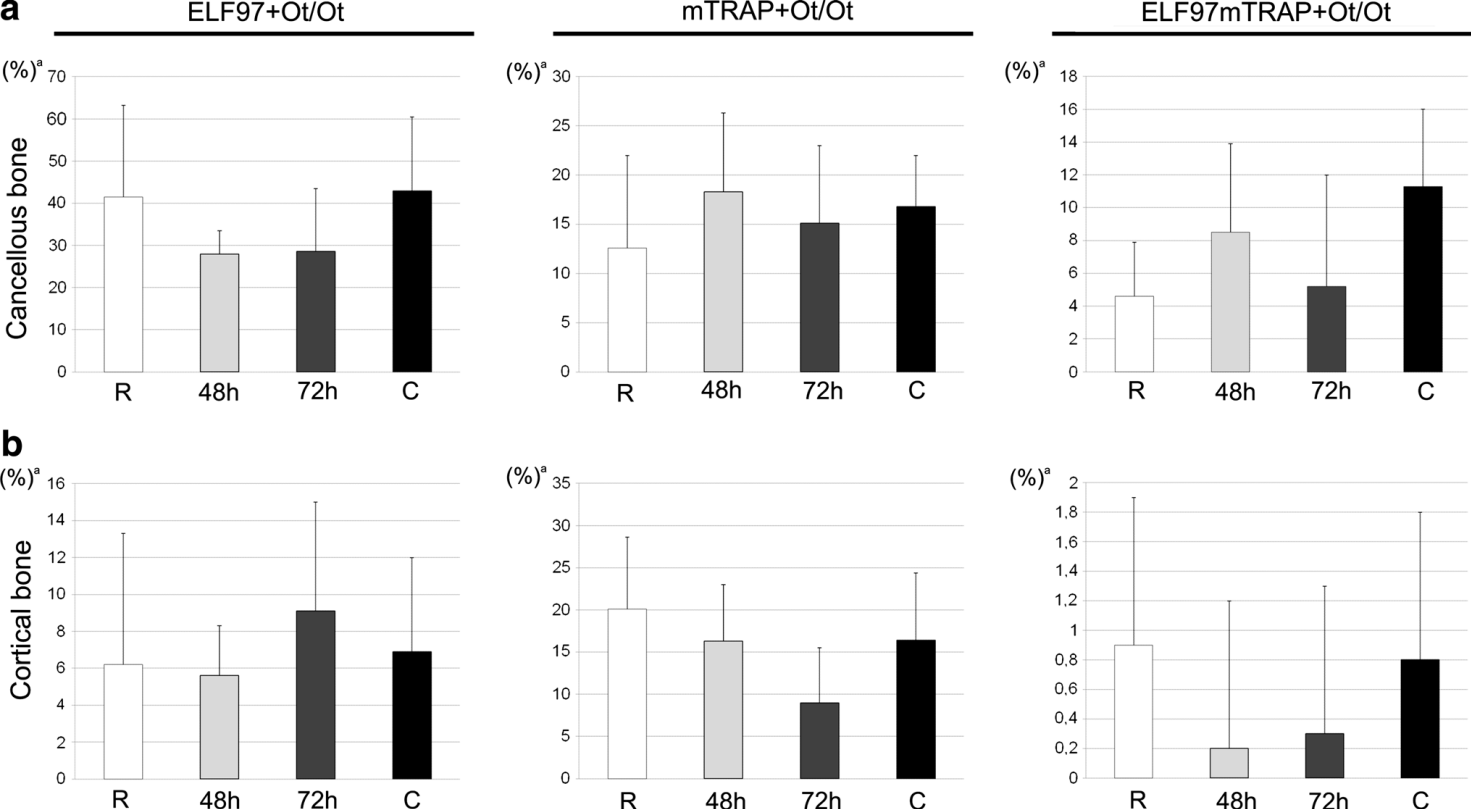
TRAP enzyme activity in osteocytes (*Ot*) in **a** cancellous and **b** cortical bone in the experimental rickets model demonstrated no significant differences between the groups; fulminant rickets (*R*), healing for 48 h, healing for 72 h, and controls (*C*) (Kruskali–Wallis test, *n* = 7 for all). ^a^ Results are presented with mean and SD

No difference was detected in TRAP enzyme activity (ELF97^+^ Ot/Ot), mTRAP^+^ Ot/Ot or ELF97mTRAP^+^ Ot/Ot in either cancellous or cortical bone in any of the groups in the experimental rickets model (Fig. 6). TRAP^+^ vesicles were observed in both osteoblasts and osteocytes in cancellous bone and presented similar features as in osteoblasts and osteocytes in the experimental osteoporosis model. However, there was no difference in the ratio TRAPv.Ar/Cy.Ar between the groups. In cortical bone only a few TRAP^+^ vesicles were observed in osteocytes and none in osteoblasts. TRAP mRNA in situ hybridization in the femoral diaphysis failed to demonstrate TRAP mRNA^+^ osteocytes in any of the groups, despite staining in the metaphyseal osteoclasts used as positive controls (data not shown) and with no staining for the sense probe.

### TRAP^+^ Vesicles Are More Abundant in Osteocytes Compared with Osteoblasts

TEM revealed increased TRAPv.Ar/Cy.Ar ratios in osteocytes versus osteoblasts in cancellous bone for animals in the experimental rickets model: *p* < 0.05 (rickets and controls), *p* < 0.01 (healing after 48 h) (Online Resource 3a). In cortical bone, osteocytes from Ovx-D and sham rats also demonstrated increased TRAPv.Ar/Cy.Ar ratios versus osteoblasts, significant in sham, *p* < 0.001 (Online Resource 3b). Only a small number of TRAP^+^ vesicles were detected in cortical osteocytes and none in cortical osteoblasts in the experimental rickets model.

### TRAP Activity in Osteoblasts and Osteocytes Is Most Prominent in Cancellous Bone

TRAP enzyme activity in osteocytes (ELF97^+^ Ot/Ot) was increased in cancellous versus cortical bone in all groups from both animal models, *p* < 0.01 (Ovx-D, sham, rickets, healing after 48 h, and controls), *p* < 0.05 (healing after 72 h) (Online Resource 4a). The vesicle ratio TRAPv.Ar/Cy.Ar was increased in cancellous versus cortical bone in osteoblasts and osteocytes in all animals in the experimental rickets groups: Ot, *p* < 0.001 (rickets and healing after 48 h), *p* < 0.05 (healing after 72 h), *p* < 0.01 (controls); Ob, *p* < 0.001 (rickets, healing after 48 h and controls), *p* < 0.01 (healing after 72 h) (Online Resource 4b, c).

### TRAP^+^ Osteocytes Are Close to Bone Surface or Bone Remodeling Surfaces

TRAP mRNA^+^ osteocytes in cortical bone and osteocytes demonstrating mTRAP and ELF97 positivity in cancellous and cortical bone were found in areas closely related to the bone surface or intracortical remodeling surfaces. The mean distance from TRAP mRNA^+^ osteocytes to the bone surface or intracortical remodeling surfaces was 33 μm, and all TRAP mRNA^+^ osteocytes were found within 148 μm from the bone surface or intracortical remodeling surfaces. mTRAP^+^ osteocytes and ELF97^+^ osteocytes in metaphyseal bone were 24 and 16 μm away from the bone surface (mean values) and no farther away than 104 and 116 μm, respectively. In cortical bone the mean distances were 60 and 27 μm, respectively, and all the TRAP^+^ osteocytes were within 166 and 113 μm, respectively. However, as these semiquantitative estimations were performed on two-dimensional sections, we cannot exclude that the real distance from the TRAP^+^ osteocytes to the bone surface or bone remodeling surfaces was shorter in another direction.

### No Difference in Osteocyte Lacunar Area in Cortical Bone in Ovx-D versus Sham

**Fig. 7:**
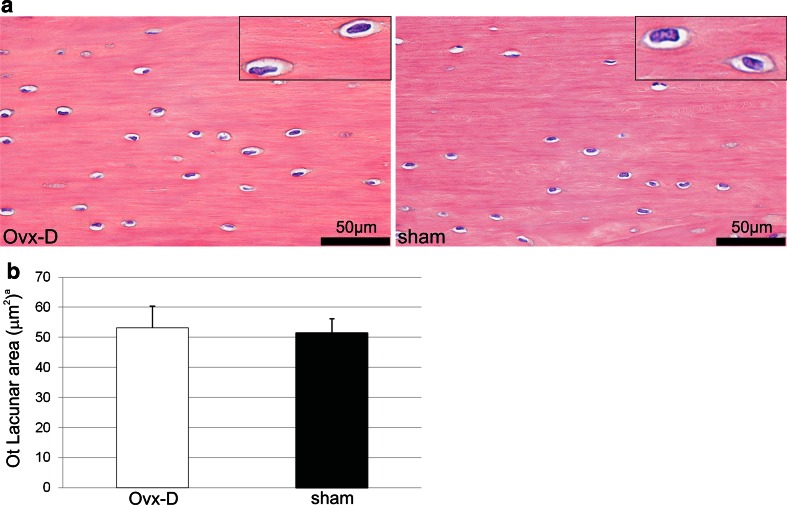
**a** Cortical bone sections (tibial diaphyses) from Ovx-D and sham animals were formalin-fixed, paraffin-embedded, and cut in 3-μm-thick sections before conventional HES staining was performed. A clear definition of the osteocyte lacunar outline was obtained after staining of tissue sections for both Ovx-D and sham. This is demonstrated by the high-power images inserted. **b** There was no difference in the osteocyte (*Ot*) lacunar area between Ovx-D and sham animals (Student’s *t* test, *n* = 7 for both). ^a^ Results are presented with mean and SD

To elucidate whether the increased level of TRAP in osteocytes in Ovx-D rats could be related to increased local resorption as described for lactating mice [19], osteocyte lacunar area in cortical bone was measured; but no difference was detected between the groups (Fig. 7).

## Discussion

Osteoclastic TRAP has been demonstrated in transcytotic intracellular vesicles as well as in the ruffled border beneath the osteoclast during active bone resorption [7, 23, 29, 35, 36]. The secretion of TRAP from the osteoclast to the resorption lacuna makes TRAP available for other bone cells, and TRAP has been suggested as one of the “coupling factors” between bone resorption and bone formation [37]. Previous studies have demonstrated TRAP in osteoblasts and osteocytes in areas close to active bone resorption sites [12, 16, 21], which has led to the hypothesis that osteoblasts and/or osteocytes engulf osteoclastic TRAP for inactivation [17]. We tested this hypothesis in vivo by analyzing TRAP expression in osteoblasts and osteocytes in two animal models with increased osteoclast activity: the experimental osteoporotic rat and rats healing from diet-induced rickets. In both models osteocytes and osteoblasts in cancellous bone and in cortical bone close to intracortical remodeling sites and endosteal/periosteal surfaces demonstrated TRAP gene expression and translation to protein as well as TRAP enzyme activity, no farther away than 166 μm. This is in line with the observations made by Nakano et al. [16], who demonstrated TRAP^+^ osteocytes in rat bone in the range of 200 μm from the resorption surfaces and concluded that there is a close relation between TRAP^+^ osteocytes and bone resorption. TEM analyses revealed TRAP in intracellular vesicles with identical morphological features in osteoblasts and osteocytes in all animals in both experimental models. This observation indicates that TRAP in osteoblasts and osteocytes might be located to endosomes, involved in intracellular transport, or stored in vesicular compartments for secretion. Moreover, the Ovx-D group demonstrated an increased ratio of TRAP^+^ vesicles in osteoblasts in cortical bone and increased TRAP enzyme activity in osteocytes in cancellous bone, and we also observed a tendency to enhanced levels of TRAP gene expression in osteocytes in cortical bone. These results indicate that the observed increase in TRAP protein expression and enzyme activity in osteoblasts and osteocytes is due to increased synthesis and not increased osteoclast activity. Moreover, no changes were demonstrated between the animals in the different groups in the experimental rickets model regarding TRAP in osteoblasts and osteocytes, despite the increased osteoclast activity in rats healing for 72 h. Thus, enhanced osteoclast activity does not change TRAP expression in vivo in osteoblasts or osteocytes in our models. Consequently, the TRAP observed in osteoblasts and osteocytes is not engulfed osteoclastic TRAP but rather synthesized in the respective cells. The theory of osteoblast and osteocyte endocytosis of osteoclastic TRAP [17] is therefore not supported by our results. A similar conclusion has been drawn by Bonucci et al. [13]: they observed that the increased level of TRAP^+^ osteoblasts in calcium-depleted rats returned to normal when calcium was replaced despite unchanged levels of TRAP^+^ osteoclasts.

Comparison of TRAP expression in cancellous versus cortical bone demonstrated enhanced levels of TRAP enzyme activity in osteocytes in all animal groups as well as an increase in the ratio of TRAP^+^ vesicles in osteoblasts and osteocytes in animals in the experimental rickets groups. There is an obvious structural difference between cancellous and cortical bone, and cancellous bone appears to be more metabolically active than cortical bone with a higher bone turnover. This might be explained by a greater surface to volume ratio in cancellous versus cortical bone [38]. The increase in TRAP^+^ vesicles and enzyme activity in osteoblasts and osteocytes in cancellous bone might therefore be linked to bone turnover; however, the mechanism remains elusive.

Qing et al. [19] observed increased TRAP as well as cathepsin K in osteocytes in lactating mice with a corresponding increase in the osteocyte lacunar area. In a recent study Kogawa et al. [39] showed that sclerostin increases the expression of TRAP, cathepsin K, and carbonic anhydrase (CA2) in osteocytes with a resulting increase in the osteocyte lacunar area. The effect is reversed by the CA2 inhibitor acetozolamide, which indicates that the osteocytic osteolysis is at least partly dependent on CA2 and its response to sclerostin. However, the effects of inhibition of TRAP or cathepsin K on the osteocyte lacunar area were not reported. Taken together, both lactation and sclerostin seem to enhance TRAP expression in osteocytes as well as ostocytic osteolysis. We investigated the effect of TRAP on osteocytic osteolysis in the experimental osteoporosis model but failed to demonstrate any difference in the osteocyte lacunar area between Ovx-D and sham despite increased osteocytic TRAP in the Ovx-D animals. An explanation for this may be that we did our measurements on decalcified tissue sections. However, this method has been used by others with success [39]. Consequently, we propose that osteocytic TRAP is not solely related to osteocytic osteolysis but has an additional role in osteocytes. Our TEM observations of TRAP located to intracellular vesicles with similar morphological features in both osteoblasts and osteocytes indicate that TRAP may have corresponding functions in the two cell types. Moreover, osteoblasts do not normally dissolve bone mineral, and the observed increase in osteoblastic TRAP in the Ovx-D animals is therefore unlikely to be related to local mineral handling by the osteoblasts. In conclusion, the role of TRAP in osteoblasts and osteocytes remains elusive. However, our results support the notion that TRAP may have another, not yet clarified role in osteocytes, in addition to the suggested contribution in local mineral handling. Results by our group indicate that the TRAP^+^ vesicles observed in osteoblasts and osteocytes also contain RANKL and OPG [40]. This may explain why only osteoblasts and osteocytes in specific areas close to the bone surface or bone remodeling surfaces demonstrate TRAP synthesis and/or enzyme activity. We therefore propose that the function of TRAP in osteoblasts and osteocytes involves the capability of the enzyme to regulate, e.g., phosphorylation of proteins known to be expressed by these cells with effects on RANKL and OPG.

## Electronic supplementary material

Below is the link to the electronic supplementary material.

Supplementary material 1 (DOCX 14 kb)

Supplementary material 2 (TIFF 28278 kb)

Supplementary material 3 (TIFF 9966 kb)

Supplementary material 4 (TIFF 444 kb)

Supplementary material 5 (TIFF 587 kb)

## References

[1] Ek-Rylander B, Barkhem T, Ljusberg J, Ohman L, Andersson KK, Andersson G (1997). Comparative studies of rat recombinant purple acid phosphatase and bone tartrate-resistant acid phosphatase. Biochem J.

[2] Fagerlund KM, Ylipahkala H, Tiitinen SL, Janckila AJ, Hamilton S, Maentausta O, Vaananen HK, Halleen JM (2006). Effects of proteolysis and reduction on phosphatase and ROS-generating activity of human tartrate-resistant acid phosphatase. Arch Biochem Biophys.

[3] Ljusberg J, Wang Y, Lang P, Norgard M, Dodds R, Hultenby K, Ek-Rylander B, Andersson G (2005). Proteolytic excision of a repressive loop domain in tartrate-resistant acid phosphatase by cathepsin K in osteoclasts. J Biol Chem.

[4] Janckila AJ, Takahashi K, Sun SZ, Yam LT (2001). Tartrate-resistant acid phosphatase isoform 5b as serum marker for osteoclastic activity. Clin Chem.

[5] Ek-Rylander B, Flores M, Wendel M, Heinegard D, Andersson G (1994). Dephosphorylation of osteopontin and bone sialoprotein by osteoclastic tartrate-resistant acid phosphatase: modulation of osteoclast adhesion in vitro. J Biol Chem.

[6] Ek-Rylander B, Andersson G (2010). Osteoclast migration on phosphorylated osteopontin is regulated by endogenous tartrate-resistant acid phosphatase. Exp Cell Res.

[7] Vaaraniemi J, Halleen JM, Kaarlonen K, Ylipahkala H, Alatalo SL, Andersson G, Kaija H, Vihko P, Vaananen HK (2004). Intracellular machinery for matrix degradation in bone-resorbing osteoclasts. J Bone Miner Res.

[8] Halleen JM, Ylipahkala H, Alatalo SL, Janckila AJ, Heikkinen JE, Suominen H, Cheng S, Vaananen HK (2002). Serum tartrate-resistant acid phosphatase 5b, but not 5a, correlates with other markers of bone turnover and bone mineral density. Calcif Tissue Int.

[9] Hayman AR, Jones SJ, Boyde A, Foster D, Colledge WH, Carlton MB, Evans MJ, Cox TM (1996). Mice lacking tartrate-resistant acid phosphatase (Acp 5) have disrupted endochondral ossification and mild osteopetrosis. Development.

[10] Suter A, Everts V, Boyde A, Jones SJ, Lullmann-Rauch R, Hartmann D, Hayman AR, Cox TM, Evans MJ, Meister T, von Figura K, Saftig P (2001). Overlapping functions of lysosomal acid phosphatase (LAP) and tartrate-resistant acid phosphatase (Acp5) revealed by doubly deficient mice. Development.

[11] Angel NZ, Walsh N, Forwood MR, Ostrowski MC, Cassady AI, Hume DA (2000). Transgenic mice overexpressing tartrate-resistant acid phosphatase exhibit an increased rate of bone turnover. J Bone Miner Res.

[12] Bianco P, Ballanti P, Bonucci E (1988). Tartrate-resistant acid phosphatase activity in rat osteoblasts and osteocytes. Calcif Tissue Int.

[13] Bonucci E, Mocetti P, Silvestrini G, Ballanti P, Zalzal S, Fortin M, Nanci A (2001). The osteoblastic phenotype in calcium-depleted and calcium-repleted rats: a structural and histomorphometric study. J Electron Microsc (Tokyo).

[14] Gradin P, Hollberg K, Cassady AI, Lang P, Andersson G (2012). Transgenic overexpression of tartrate-resistant acid phosphatase is associated with induction of osteoblast gene expression and increased cortical bone mineral content and density. Cells Tissues Organs.

[15] Mocetti P, Ballanti P, Zalzal S, Silvestrini G, Bonucci E, Nanci A (2000). A histomorphometric, structural, and immunocytochemical study of the effects of diet-induced hypocalcemia on bone in growing rats. J Histochem Cytochem.

[16] Nakano Y, Toyosawa S, Takano Y (2004). Eccentric localization of osteocytes expressing enzymatic activities, protein, and mRNA signals for type 5 tartrate-resistant acid phosphatase (TRAP). J Histochem Cytochem.

[17] Perez-Amodio S, Jansen DC, Tigchelaar-Gutter W, Beertsen W, Everts V (2006). Endocytosis of tartrate-resistant acid phosphatase by osteoblast-like cells is followed by inactivation of the enzyme. Calcif Tissue Int.

[18] Perez-Amodio S, Vogels IM, Schoenmaker T, Jansen DC, Alatalo SL, Halleen JM, Beertsen W, Everts V (2005). Endogenous expression and endocytosis of tartrate-resistant acid phosphatase (TRACP) by osteoblast-like cells. Bone.

[19] Qing H, Ardeshirpour L, Pajevic PD, Dusevich V, Jahn K, Kato S, Wysolmerski J, Bonewald LF (2012). Demonstration of osteocytic perilacunar/canalicular remodeling in mice during lactation. J Bone Miner Res.

[20] Wergedal JE, Baylink DJ (1969). Distribution of acid and alkaline phosphatase activity in undemineralized sections of the rat tibial diaphysis. J Histochem Cytochem.

[21] Yamamoto T, Nagai H (1998). Ultrastructural localization of tartrate-resistant acid phosphatase activity in rat osteoblasts. J Electron Microsc (Tokyo).

[22] Melhus G, Solberg LB, Dimmen S, Madsen JE, Nordsletten L, Reinholt FP (2007). Experimental osteoporosis induced by ovariectomy and vitamin D deficiency does not markedly affect fracture healing in rats. Acta Orthop.

[23] Hollberg K, Nordahl J, Hultenby K, Mengarelli-Widholm S, Andersson G, Reinholt FP (2005). Polarization and secretion of cathepsin K precede tartrate-resistant acid phosphatase secretion to the ruffled border area during the activation of matrix-resorbing clasts. J Bone Miner Metab.

[24] Dempster DW, Compston JE, Drezner MK, Glorieux FH, Kanis JA, Malluche H, Meunier PJ, Ott SM, Recker RR, Parfitt AM (2013). Standardized nomenclature, symbols, and units for bone histomorphometry: a 2012 update of the report of the ASBMR Histomorphometry Nomenclature Committee. J Bone Miner Res.

[25] National Research Coucil (2011). Guide for the care and use of laboratory animals.

[26] Hultenby K, Reinholt FP, Oldberg A, Heinegard D (1991). Ultrastructural immunolocalization of osteopontin in metaphyseal and cortical bone. Matrix.

[27] Rissanen JP, Suominen MI, Peng Z, Halleen JM (2008). Secreted tartrate-resistant acid phosphatase 5b is a marker of osteoclast number in human osteoclast cultures and the rat ovariectomy model. Calcif Tissue Int.

[28] Ek-Rylander B, Bill P, Norgard M, Nilsson S, Andersson G (1991). Cloning, sequence, and developmental expression of a type 5, tartrate-resistant, acid phosphatase of rat bone. J Biol Chem.

[29] Melhus G, Brorson SH, Baekkevold ES, Andersson G, Jemtland R, Olstad OK, Reinholt FP (2010). Gene expression and distribution of key bone turnover markers in the callus of estrogen-deficient, vitamin D-depleted rats. Calcifi Tissue Int.

[30] Zenger S, Ek-Rylander B, Andersson G (2010). Biogenesis of tartrate-resistant acid phosphatase isoforms 5a and 5b in stably transfected MDA-MB-231 breast cancer epithelial cells. Biochim Biophys Acta.

[31] Filgueira L (2004). Fluorescence-based staining for tartrate-resistant acidic phosphatase (TRAP) in osteoclasts combined with other fluorescent dyes and protocols. J Histochem Cytochem.

[32] Lang P, Andersson G (2005). Differential expression of monomeric and proteolytically processed forms of tartrate-resistant acid phosphatase in rat tissues. Cell Mol Life Sci.

[33] Schneider CA, Rasband WS, Eliceiri KW (2012). NIH Image to ImageJ: 25 years of image analysis. Nat Methods.

[34] Brorson SH, Roos N, Skjorten F (1994). Antibody penetration into LR-white sections. Micron.

[35] Nordahl J, Andersson G, Reinholt FP (1998). Chondroclasts and osteoclasts in bones of young rats: comparison of ultrastructural and functional features. Calcif Tissue Int.

[36] Nordahl J, Hollberg K, Mengarelli-Widholm S, Andersson G, Reinholt FP (2000). Morphological and functional features of clasts in low phosphate, vitamin D-deficiency rickets. Calcif Tissue Int.

[37] Sheu TJ, Schwarz EM, Martinez DA, O’Keefe RJ, Rosier RN, Zuscik MJ, Puzas JE (2003). A phage display technique identifies a novel regulator of cell differentiation. J Biol Chem.

[38] Parfitt AM (2002). Misconceptions (2): turnover is always higher in cancellous than in cortical bone. Bone.

[39] Kogawa M, Wijenayaka AR, Ormsby R, Thomas GP, Anderson PH, Bonewald LF, Findlay DM, Atkins GJ (2013). Sclerostin regulates release of bone mineral by osteocytes by induction of carbonic anhydrase 2. J Bone Miner Res.

[40] Solberg LB, Stang E, Brorson SH, Andersson G, Reinholt FP (2013) Tartrate-resistant acid phosphatase (TRAP) co-localizes with receptor activator of NF-κΒ ligand (RANKL) in osteoblasts and osteocytes: a potential role for TRAP in regulation of osteoclast differentiation. ASBMR Annual Meeting, Baltimore, October 4–7, 2013, abstract MO0286

