# Supplementary material for: Increased Tartrate-Resistant Acid Phosphatase Expression in Osteoblasts and Osteocytes in Experimental Osteoporosis in Rats
Source: Calcif Tissue Int. 2014 Jan 7;94(5):510–21. doi: 10.1007/s00223-013-9834-3 (PMC4148331; doi:10.1007/s00223-013-9834-3)
Supplement: Supplementary file 1 — Supplementary material 1 (DOCX 14 kb) [file 223_2013_9834_MOESM1_ESM.docx]

**FIGURE CAPTIONS FOR ONLINE RESOURCES**

**Online Resource 1** Animal models **a** Light microscopic images display a large difference in trabecular bone volume (BV/TV) between Ovx-D and sham. **b** BV/TV in femoral head and bone mineral density (BMD) in femoral neck and vertebrae were decreased in Ovx-D vs. sham (Student’s t-test, n=7/7). **c** The animals with fulminant rickets demonstrated enlarged physis/metaphysis and lack of well-defined EMB, while a healing zone (He.Z) had developed at 48h healing. After healing for 72h the animals showed almost a normal structure at the EMB but still with a small He.Z compared to controls. ^a^The results are presented with mean and SD, ^b^The results are means of the right and left limb, *** p<0.001

**Online Resource 2** TRAP enzyme activity and monomeric TRAP (mTRAP) immunolabeling in osteocytes (Ot) in cortical bone from femur diaphysis. **a** HES stained sections of cortical bone in sham and **e** Ovx-D show the tissue architecture. The black outlines demonstrate corresponding areas to the immunofluorescence images. **b-d** ELF97+ Ot (yellow-green, arrows), mTRAP+ Ot (red) and ELF97mTRAP+ Ot (arrowheads) in sham and **f-h** Ovx-D. **i** Inhibition of TRAP enzyme activity with molybdate demonstrated low background fluorescence for ELF97. **j** Unspecific rabbit IgG served as negative control for mTRAP with low background fluorescence. Scale-bars 10µm

**Online Resource 3** Histomorphometric semi-quantitative analyses of TRAPv.Ar/Cy.Ar in osteocytes (Ot) and osteoblasts (Ob). **a** TRAPv.Ar/Cy.Ar Ot vs. Ob in cancellous bone in experimental rickets (n=7/7/6/7). **b** TRAPv.Ar/Cy.Ar Ot vs. Ob in cortical bone in all groups (n=7/7/7/7/5/6) (b). The statistical analyses are performed with Mann-Whitney test. ^a^The results are presented with mean and SD, ** p<0.01, *** p<0.001

**Online Resource 4** TRAP expression increased in osteocytes (Ot) and osteoblasts (Ob) in cancellous vs. cortical bone. **a** TRAP enzyme activity in all groups displayed by ELF97+Ot/Ot. **b** TRAPv.Ar/Cy.Ar in Ot in experimental rickets. **c** TRAPv.Ar/Cy.Ar in Ob in experimental rickets. The statistical analyses are performed with Mann-Whitney test. ^a^The results are presented with mean and SD, * p<0.05, ** p<0.01, *** p<0.001
