# Supplementary figures and images for: Increased Tartrate-Resistant Acid Phosphatase Expression in Osteoblasts and Osteocytes in Experimental Osteoporosis in Rats
Source: Calcif Tissue Int. 2014 Jan 7;94(5):510–21. doi: 10.1007/s00223-013-9834-3 (PMC4148331; doi:10.1007/s00223-013-9834-3)

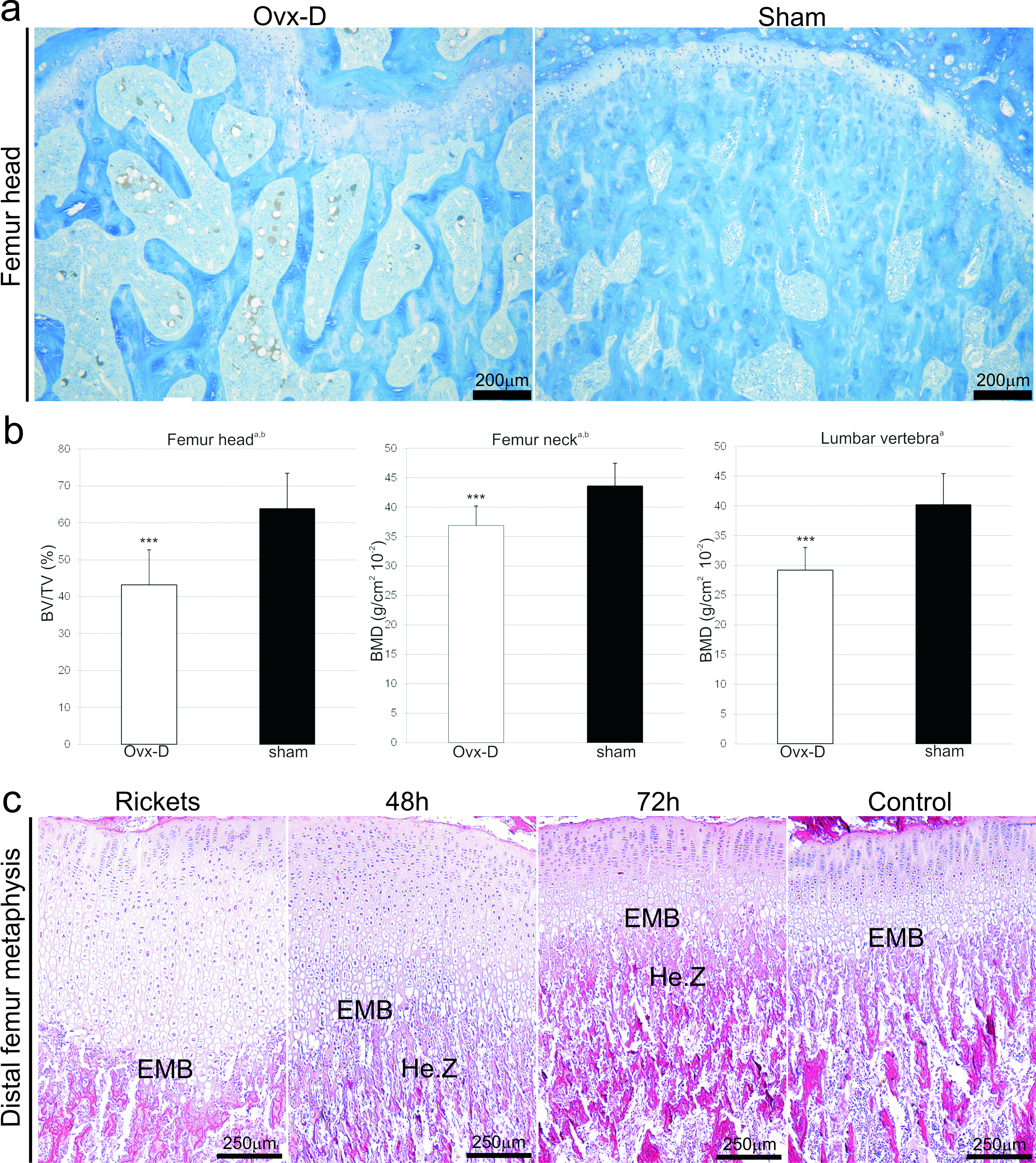

Supplement: Supplementary file 2 — Supplementary material 2 (TIFF 28278 kb) [file 223_2013_9834_MOESM2_ESM.tif]

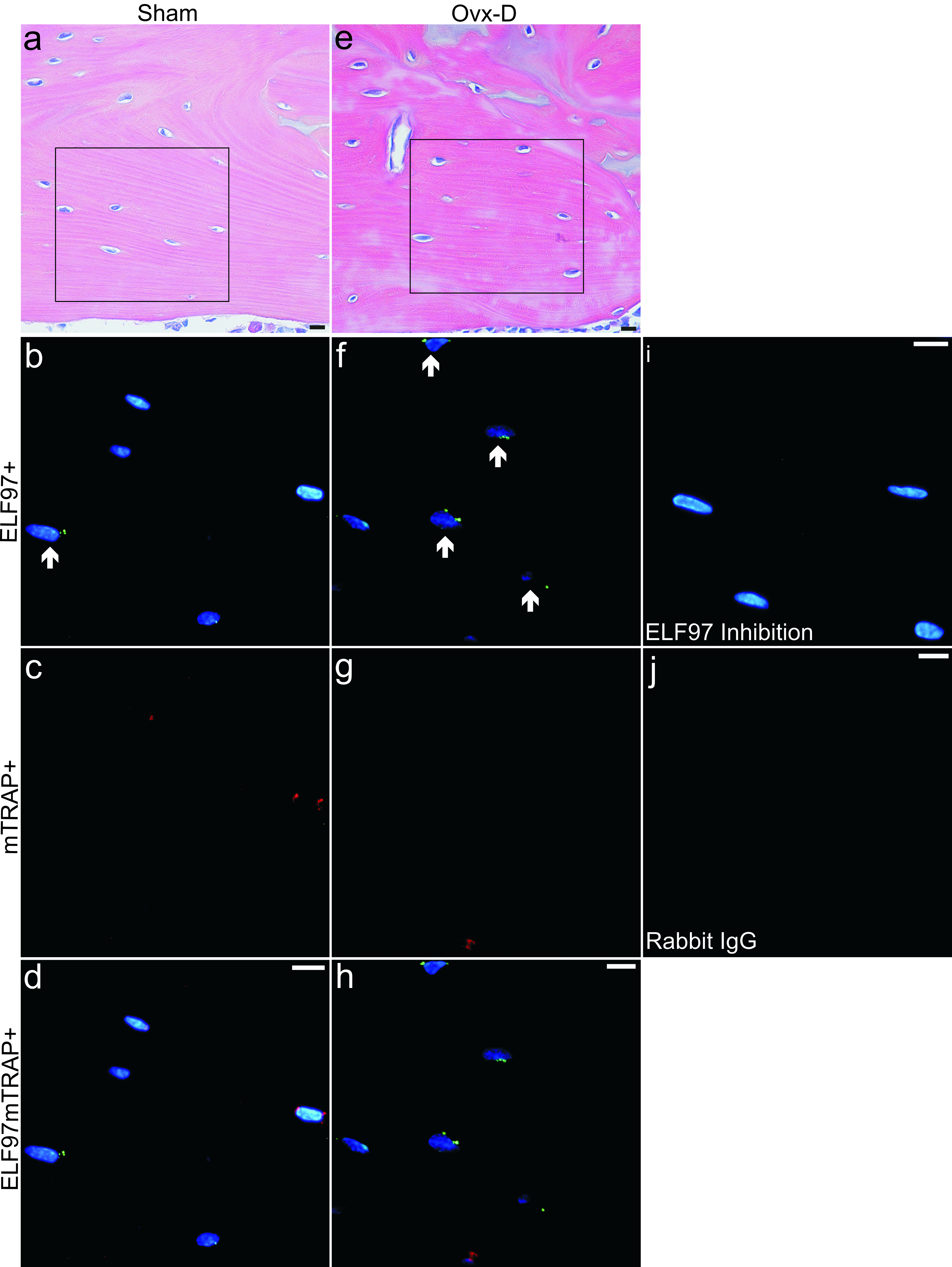

Supplement: Supplementary file 3 — Supplementary material 3 (TIFF 9966 kb) [file 223_2013_9834_MOESM3_ESM.tif]

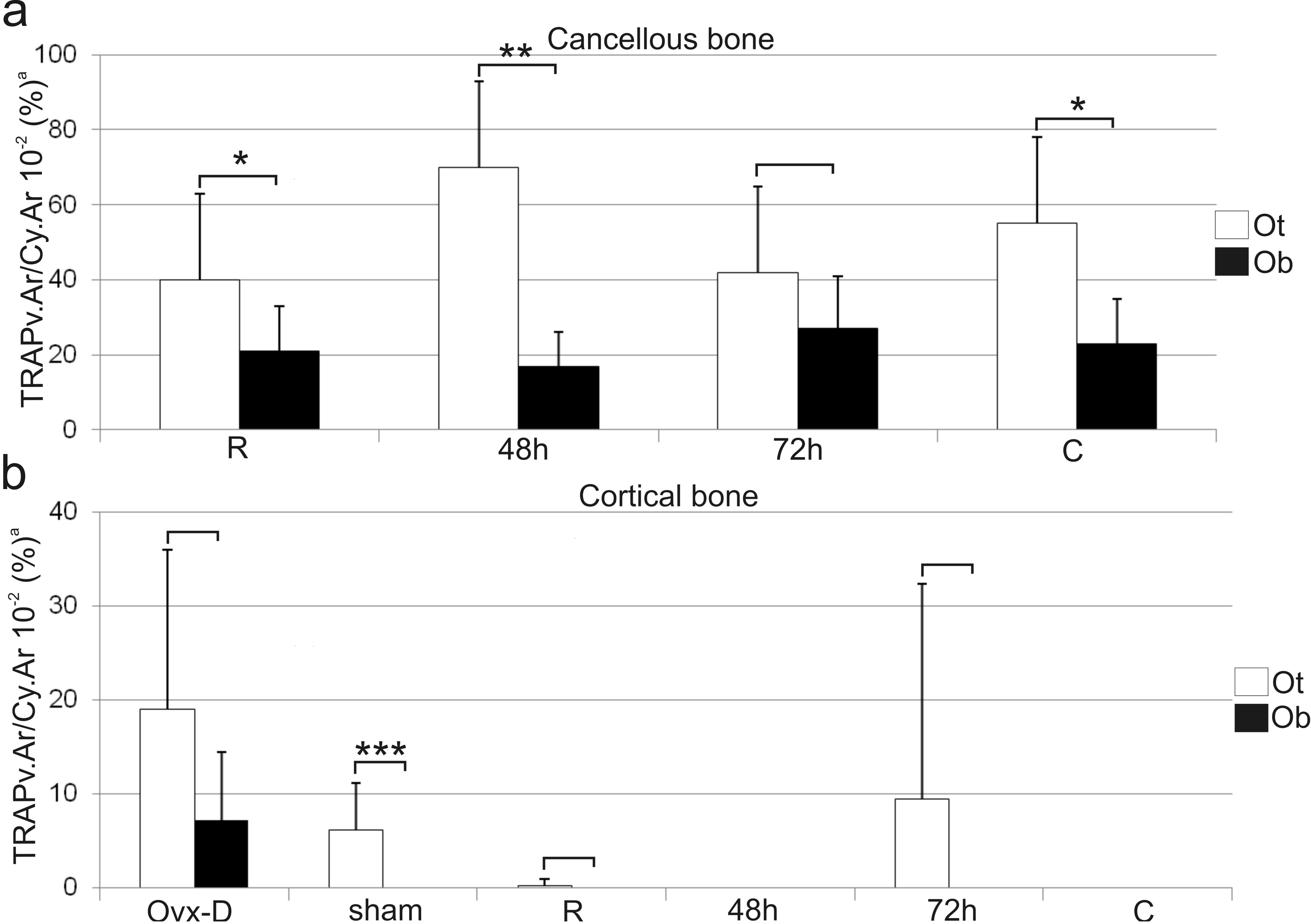

Supplement: Supplementary file 4 — Supplementary material 4 (TIFF 444 kb) [file 223_2013_9834_MOESM4_ESM.tif]

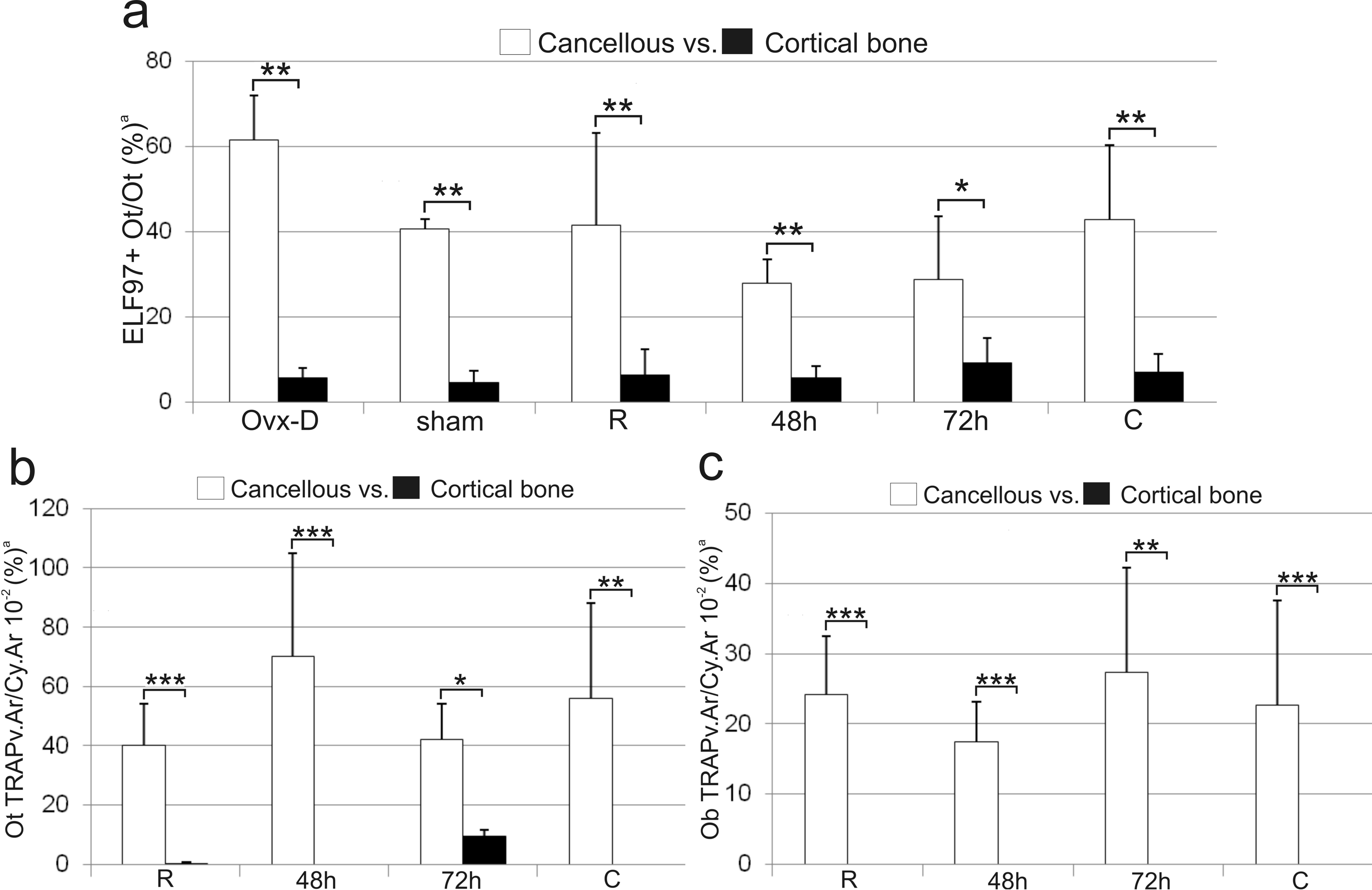

Supplement: Supplementary file 5 — Supplementary material 5 (TIFF 587 kb) [file 223_2013_9834_MOESM5_ESM.tif]
